# Conversion of Solid Organic Wastes into Oil via *Boettcherisca peregrine* (Diptera: Sarcophagidae) Larvae and Optimization of Parameters for Biodiesel Production

**DOI:** 10.1371/journal.pone.0045940

**Published:** 2012-09-24

**Authors:** Sen Yang, Qing Li, Qinglan Zeng, Jibin Zhang, Ziniu Yu, Ziduo Liu

**Affiliations:** 1 State Key Laboratory of Agricultural Microbiology, National Engineering Research Centre of Microbial Pesticides, College of Life Science and Technology, Huazhong Agricultural University, Wuhan, Hubei, People’s Republic of China; 2 College of Science, Huazhong Agricultural University, Wuhan, Hubei, People’s Republic of China; 3 Department of Biological Engineering, Xianning Vocational Technical College, Xianning, China; University of Nottingham, United Kingdom

## Abstract

The feedstocks for biodiesel production are predominantly from edible oils and the high cost of the feedstocks prevents its large scale application. In this study, we evaluated the oil extracted from *Boettcherisca peregrine* larvae (BPL) grown on solid organic wastes for biodiesel production. The oil contents detected in the BPL converted from swine manure, fermentation residue and the degreased food waste, were 21.7%, 19.5% and 31.1%, respectively. The acid value of the oil is 19.02 mg KOH/g requiring a two-step transesterification process. The optimized process of 12∶1 methanol/oil (mol/mol) with 1.5% H_2_SO_4_ reacted at 70°C for 120 min resulted in a 90.8% conversion rate of free fatty acid (FFA) by esterification, and a 92.3% conversion rate of triglycerides into esters by alkaline transesterification. Properties of the BPL oil-based biodiesel are within the specifications of ASTM D6751, suggesting that the solid organic waste-grown BPL could be a feasible non-food feedstock for biodiesel production.

## Introduction

With economic development and population growth, large quantities of solid organic wastes such as animal waste (e.g., manure), food waste and fermentation industry waste (e.g., filter cake, etc.) are generated all over the world, especially in developing countries. These wastes, if not properly managed, will not only cause environmental pollution but also resource wasting. On the other hand, the reserves of conventional energies are non-renewable, and the major energy resources for almost every country are progressively decreasing and predicted to exhaust in the near future [1]. Biodiesel, one of the recyclable energies, has been considered an ideal substitute for fossil fuels. Currently, about 84% of the world’s biodiesel is produced from rapeseed oil, followed by sunflower oil (13%), palm oil (1%), and soybean oil and others (2%) [2]. However, a large-scale biodiesel production from edible oils can bring a global imbalance to the food supply and demand market, and lead to deforestation and destruction of ecosystem on the planet [3], [4]. Therefore, a promising solution to environmental and energy crises is to use non-edible oils, such as insect grease/oil derived from solid organic wastes [5].

Insects, one of the largest biomass in the world, could be found nearly in every corner of the earth, and insect fat/oil is igniting particular interest among researchers [6], [7], [8]. Many saprophagous insect larvae fed on solid organic wastes (e.g., animal manure, food waste etc.) can be converted into insect oil and nutrition [5]. For example, black soldier fly (*Hermetia illucens*) larvae converted from organic wastes (cattle manure, pig manure, and chicken manure) can be used as a resource for biodiesel production [9]. Besides, black soldier fly can also help reduce the solid residual fraction (hereafter SRF) accumulation of restaurant waste after typical grease extraction, and increase the overall biodiesel yield [10]. Li et al. reported that 5-days *Chrysomya megacephala* (Fabricius) larvae fed on restaurant garbage could be used as a raw material for biodiesel production [11]. However, little information is currently available on the use of *Boettcherisca peregrine* (flesh fly) larvae as an alternative biodiesel feedstock.

*B. peregrine* belongs to the Boettcherisca genus, Sarcophagidae family, and Diptera order, and is widely used as a death investigator in forensic entomology [12], with a wide geographic distribution from semitropical to tropical regions. Its life cycle comprises of three stages: larva (2–3 days), pupa (8–10 days) and adult at a temperature between 25–30°C [13], [14]. Solid organic wastes such as animal manure and carrion are the main breeding grounds for *B. peregrine* larvae (hereafter BPL) and cattle liver can be used to establish a laboratory colony [15]. Occasionally, BPL are found to grow thriftily on the decayed gibberellin A3 fermentation residue (hereafter GFR), a by-product derived from *Fusarium fujikuroi* fermentation liquor in gibberellin A3 production [16], but *C. megacephala* larvae can not develop normally on GFR.

In this paper, we report the use of GFR, swine manure and SRF as media for rearing BPL and the extraction of oil from 4-days BPL for biodiesel production. Additionally, we optimized four variables affecting the yield of acid-catalyzed production of methyl esters: the molar ratio of methanol to oil, catalyst amount, reaction time and temperature. The major fatty acid components of the BPL oil-based biodiesel were also compared with those of three known biodiesels. Finally, the properties of the produced fatty acid methyl esters (hereafter FAME) were evaluated against ASTM D6751, aiming to explore the feasibility of biodiesel production from BPL fed on solid organic wastes.

## Materials and Methods

### Collection of Fly Source and Solid Organic Wastes

The initial *B. peregrine* larvae were gathered from the swine manure at the National Engineering Research Centre of Microbial Pesticides, Huazhong Agricultural University (at 29–31°N, 113–115°E). The larvae were reared with a medium made of wheat bran and fish meal (7∶3, w/w) before the mature larvae were collected from the digested medium in a cage for eclosion. The adults were kept at a constant temperature, humidity and photoperiod (25±5°C, 70±5% RH, 12L:12D) in experimental cages (50×50×50 cm) and provided ad libitum with water and food (brown sugar: milk powder = 1∶1). Decayed fish meal (65% moisture) was used as an oviposition substrate to induce the adults to produce larvae. This laboratory colony was established during the spring of 2011, and has been maintained for more than 10 generations.

GFR was a gift from Jiang Xi New Reyphon Biochemical Co., LTD, which contained 60% water, 25% crude protein, 14.9% crude fat, 7.0% crude fiber, 5.6% crude ash, 0.9% phosphorus, 0.5% calcium and 0.3% potassium (dry matter base, w/w). Swine manure was collected from the Pig Breeding Farm of Huazhong Agricultural University, which contained 76% water, 17.5% crude protein, 13.8% crude fat, 16.0% crude fiber, 12.7% crude ash, 1.5% phosphorus, 1.4% calcium and 0.5% potassium (dry matter base, w/w). Raw restaurant waste was collected from restaurants in Wuhan City, China, by Hubei Tianji Bioengineer Co. Ltd., a government authorized biodiesel processing plant located in Huazhong Agricultural University. Waste grease was extracted from the raw restaurant waste in the processing plant and used for biodiesel production. On average, about 3% (w/w) of the raw restaurant waste was separated as waste grease, which was further processed into biodiesel with an overall biodiesel yield of 2.7% (w/w) (Unpublished data from Tianji Bioengineer Co. Ltd.) [10]. The SRF of the restaurant waste after typical grease extraction was used to rear BPL, which contained 80% water, 27.8% crude protein, 11.6% crude fat, 7.4% crude fiber, 13.6% crude ash, 0.7% phosphorus, 0.8% calcium, and 1.3% potassium (dry matter base, w/w).

### Conversion of Solid Organic Wastes into Larvae

**Figure 1:**
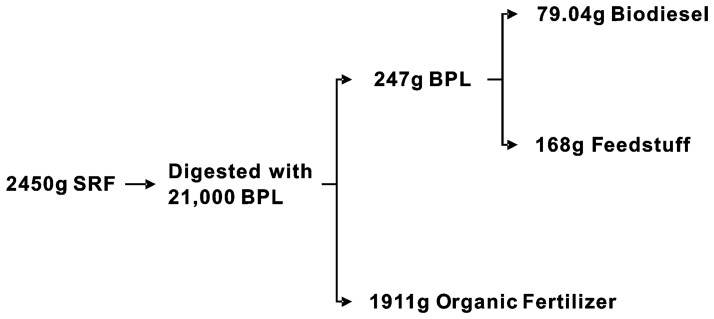
Conversion process from SRF into biodiesel via BPL.

Based on our previous studies, 40% rice straw powder (w/w, dry matter) was added into GFR to adjust its permeability for BPL feeding. Approximately 7 kg of solid organic wastes (GFR containing rice straw powder, swine manure and SRF, 65% moisture) was placed, respectively, in a plastic tank (0.65×0.43×0.14 m) to a thickness of 5 cm and exposed for biodegradation by 21,000 BPL (Fig. 1). After 4d of cultivation, the mature larvae were separated from residue by sieving, dried at 105°C and ground for oil extraction.

### Oil Extraction

BPL oil was extracted using a modified method described by Li et al [9]. The ground BPL powder was placed in a filter bag and soaked in petroleum ether (2000 ml) for 48 h at room temperature. Crude BPL oil was obtained by evaporating petroleum ether with a rotary evaporator.

### Optimization of Parameters Affecting Biodiesel Production

The acid value of the BPL oil is 19.02 mg KOH/g, which was determined by titration with the standard method (ISO 660, 1996).To improve the FFA conversion efficiency by acid-catalyzed esterification, four principal variables were investigated: reaction time, reaction temperature, catalyst amount, and the molar ratio of methanol to oil [17]. The optimization of the four parameters was performed by pretreating twenty groups with methanol using H_2_SO_4_ as the catalyst under following conditions: five groups were pretreated (1.0% catalyst, 65°C, 60 min, 200 rpm) with five different methanol to oil molar ratios of 6∶1, 8∶1, 10∶1, 12∶1 and 14∶1; five groups were pretreated (12∶1methanol to oil molar ratio, 65°C, 60 min, 200 rpm) with five different catalyst amounts of 0.5%, 1.0%, 1.5%, 2.0% and 2.5%; five groups were pretreated (1.5% catalyst, 12∶1 methanol to oil molar ratio, 60 min, 200 rpm) at five different temperatures of 60°C, 65°C, 70°C, 75°C and 80°C; five groups were pretreated (1.5% catalyst, 12∶1 methanol to oil molar ratio, 70°C, 200 rpm) for five different time periods of 30 min, 60 min, 90 min, 120 min and 150 min. Each group had 3 replicates of 10 g of crude BPL oil. The esterification apparatus consists of a water bath with a mechanical stirrer, a digital temperature controller and a 50 ml three-neck round bottom flask.

After esterification, the resulting mixture was poured into a 50 ml extraction bottle, and was separated by gravity. Subsequently, the upper layer was transferred to a reactor, and dried in an oven at 105°C for 2 hours before being mixed with methanol (8∶1methanol to oil molar ratio) and the catalyst NaOH (1%, w/w). After a 60°C water bath for 30 min with stirring, the mixture was separated by gravity. Finally, the upper biodiesel layer was separated from the lower layer with a funnel and distilled at 80°C to remove the residual methanol and water.

### Analysis

During esterification, 2 ml samples were withdrawn periodically to determine the acid value by titration. The acid value (AV) of the crude oil was determined by titration with potassium hydroxide. FFA conversion rate (%) was calculated using the following formula:
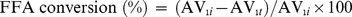
where AV*_i_* represents the initial acid value, AV*_t_* represents the acid value at reaction time *t*.

The fatty acid compositions were determined by a GC/MS (Thermo-Finnigan, USA) equipped with a polyethylene glycol phase capillary column (Agilent, USA) according to the method from EN 14103 [27]. Other biodiesel characteristics such as density (EN ISO3675), viscosity (EN3105), flash point (EN ISO3679) and cetane index (EN ISO5165) were also measured.

## Results and Discussion

### BPL Yield

**Figure 2:**
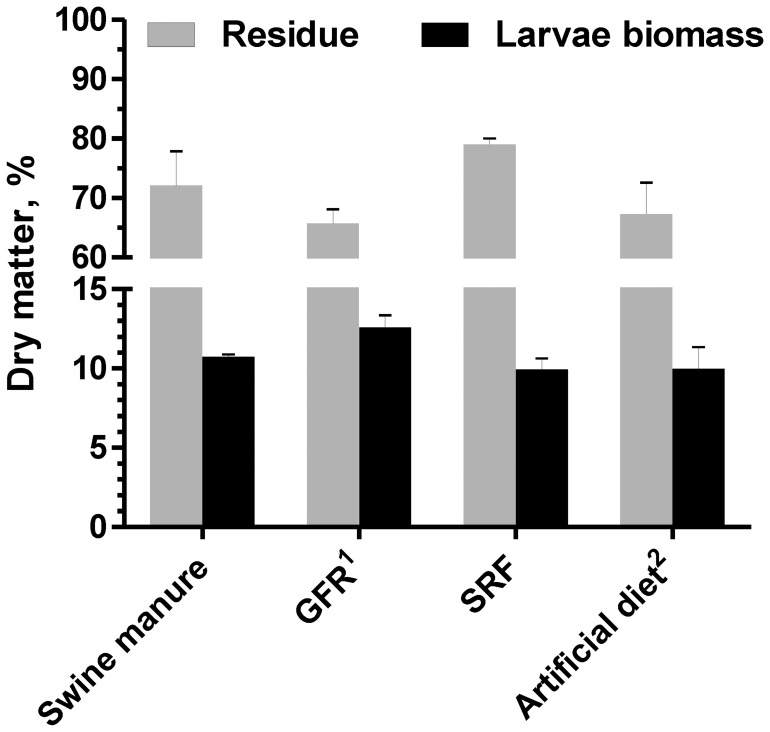
Yields of BPL biomass and residues derived from solid organic wastes. ^1^GFR: gibberellin A3 fermentation residue and rice straw powder (6∶4, w/w); ^2^artificial diet: wheat bran and fish meal (7∶3, w/w).

As shown in Fig. 1, about 21,000 BPL reduced 2450 g of SRF to 1911 g of dry digested SRF within 4 days and 247 g of dried larvae in the end. Fig. 2 presents the BPL yields and digested residue derived from four different diets: swine manure, GFR (mixed with 40% rice straw powder, w/w), SRF and artificial diet made of wheat bran and fish meal mixture (7∶3, w/w). The GFR achieved the highest yield (12.6%), followed by the swine manure (10.7%), the artificial diet (10.0%) and the SRF (9.9%), indicating that GFR could be the most efficient resource for culturing BPL. Nevertheless, the GFR influence on the growth of BPL needs further studying. The SRF achieved the lowest BPL yield, because SRF medium varied in composition [18]. Yields of digested residue ranged from 65.7% to 79.0% for those solid organic wastes.

In this study, the conversion of the solid organic wastes into BPL for oil extraction was accomplished in only 4 days, much shorter than the time for black soldier fly larvae (15 days) and *C. megacephala* (Fabricius) larvae (5 days). The possible reason is that BPL are ovoviviparous and the larva stage only lasts about 3 days under the optimal condition [13], which suggests a shorter production cycle and a lower cost in treating organic wastes with BPL on a large scale.

### Crude Oil Extraction

**Figure 3:**
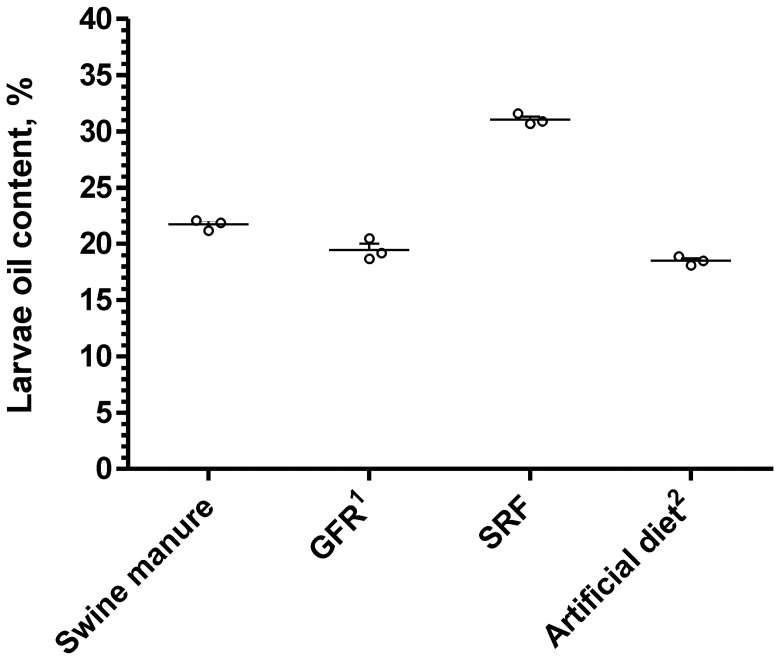
Crude oil content in BPL biomass grown on solid organic wastes.

Fig. 3 shows the crude oil content in the BPL grown on solid organic wastes (dry matter base), indicating a connection of the oil content with the larvae media. The BPL derived from SRF has the highest oil content (31.1%), slightly lower than the black soldier fly larvae fed on SRF (39.2%), but higher than the 5-days *C. megacephala* (Fabricius) larvae (24.40% to 26.29%) fed on restaurant garbage [10], [11]. The BPL converted from swine manure contains 21.7% of oil, lower than rapeseed (37%), but higher than soybean (20%) [1]. The BPL converted from GFR contains the least oil (19.5%), still higher than the oil content of okra seed (12%), the main feedstock for biodiesel production in tropical and subtropical regions [19].

### Oil Properties

Table 1 shows the properties of BPL oil. The BPL acid value is 19.02 mg KOH/g, which is close to that of kusum (*Schleichera triguga*) oil (21.30 mg KOH/g), an ideal feedstock for biodiesel production. Acid value indicates the amount of free fatty acid in oil [17]. Saponification value is used for measuring the average molecular weight of oil and expressed in milligrams of potassium hydroxide (mg KOH/g oil). BPL oil has a saponification value of 220.5 mg KOH/g oil, higher than that of *H. illucens* larvae oil (146.6 mg KOH/g oil) and *C. Megacephala* larvae oil (202.1 mg KOH/g oil), respectively [10], [11]. The iodine value of BPL oil (98.5 cg I/g oil) is higher than that of *C. Megacephala* larvae oil (73 cg I/g oil), but lower than that of rapeseed oil (108.1 cg I/g oil). Iodine value indicates the degree of unsaturation of an oil [20]. Additionally, BPL oil has a lower melting point (2.0°C), but a higher peroxide value than *H. illucens* larvae oil (4.0°C). The melting point refers to the freezing point of oil, and the peroxide value indicates the level of rancidity during storage. All the data suggest that BPL oil is suitable for biodiesel production.

**Table 1:** Properties of *B. peregrine* larvae oil and three known oils.

Properties	Units	*B. peregrine*	*H. illucens* a	*C. Megacephala* b	Rapeseed^c^
Acid value	mg KOH/g	19.02	7.1	1.10	0.20
Density	kg/m^3^	944	899	n/a	912
Saponification value	mg KOH/g	220.5	146.6	202.1	197.1
Iodine value	cg I/g oil	98.5	89.7	73.0	108.1
Melting point	°C	2.0	4	n/a	n/a
Peroxide value	meq/kg	0.2	0.04	n/a	n/a

n/a stands for not reported;

a data from literature [10];

b data from literature [11];

c data from literature [20].

**Table 2:** Fatty acid compositions of *B. peregrine* larvae oil and three known oils.

Fatty acid (%)	Structure	*B. peregrine*	*H. illucens* a	*C. Megacephala* b	Rapeseed c
Capric acid	C10:0	n/d	3.1	n/a	n/a
Lauric acid	C12:0	n/d	35.6	n/a	n/a
Myristic acid	C14:0	1.4	n/a	3.9	n/a
Pentadecanoic acid	C15:0	1.6	n/a	0.3	n/a
Palmitic acid	C16:0	18.9	14.8	35.5	3.5
Margaric acid	C17:0	0.4	n/a	0.3	n/a
Stearic acid	C18:0	0.2	3.6	2.8	0.8
Noadecanic acid	C19:0	0.5	n/a	n/a	n/a
Arachidic acid	C20:0	n/d	n/a	0.4	n/a
Myristic acid	C14:1	n/d	7.6	n/a	n/a
Palmitoletic acid	C16:1	18.3	3.8	13.0	n/a
Heptadecenoic acid	C17:1	0.4	n/a	n/a	n/a
Oleic acid	C18:1	44.5	23.6	24.4	64.4
Linoleic acid	C18:2	4.1	5.8	15.3	22.3
Linolenic acid	C18:3	7.6	n/a	1.3	n/a
Nonadecanoic acid	C19:1	n/d	1.4	n/a	n/a
Saturated fatty acid		23.2	57.1	43.2	4.3
Unsaturated fatty acid		75.1	40.8	54.6	94.9
Odd-carbon fatty acid		2.4	1.4	0.6	n/a
Total		98.3	97.9	97.8	99.2

n/d stands for not detected; n/a stands for not reported;

a data from literature [10];

b data from literature [11];

c data from literature [20].

Table 2 shows the fatty acid compositions of BPL oil compared with three different oils: *H. illucens* larvae oil, *C. Megacephala* larvae oil and rapeseed oil. 11 different fatty acids were detected and identified, and the main fatty acids identified in BPL oil were oleic acid, palmitic acid, and palmitoletic acid. In most organisms, the major acid is palmitic, followed by oleic and other acids typical of the species [21]. The concentration of oleic acid (44.5%) in BPL oil is nearly double that in *H. illucens* larvae oil (23.6%) and *C. Megacephala* larvae oil (24.4%), but lower than that in rapeseed oil (64.4%). In BPL oil, the concentration of palmitic acid (18.9%) is close to that of palmitoletic acid (18.2%). Interestingly, the concentration of total odd-carbon fatty acids (C15:0, C17:0, C19:0 and C17:1) is 2.4% in BPL oil, but only 1.4% and 0.6% in *H. illucens* larvae oil and *C. Megacephala* larvae oil, respectively. The odd-carbon fatty acids are major fatty acids in microorganisms like thraustochytrids which have the potential as a feedstock for biofuel production [22], but are typically minor (<1%) components in lower plants or animals [21]. It is reported that the fatty acid methyl esters derived from odd-carbon fatty acids have a better low temperature performance than those from even-carbon fatty acids [23]. Furthermore, BPL oil not only contains more total saturated fatty acids (23. 2%) than peanut oil (17.8%), rapeseed oil (4.4%), sunflower oil (9.3%), and soybean oil (16%), respectively [20], but also contains more total unsaturated fatty acids (75.1%) than *H. illucens* larvae oil (40.8%) and *C. Megacephala* larvae oil (54.6%), respectively [10], [11].

**Figure 4:**
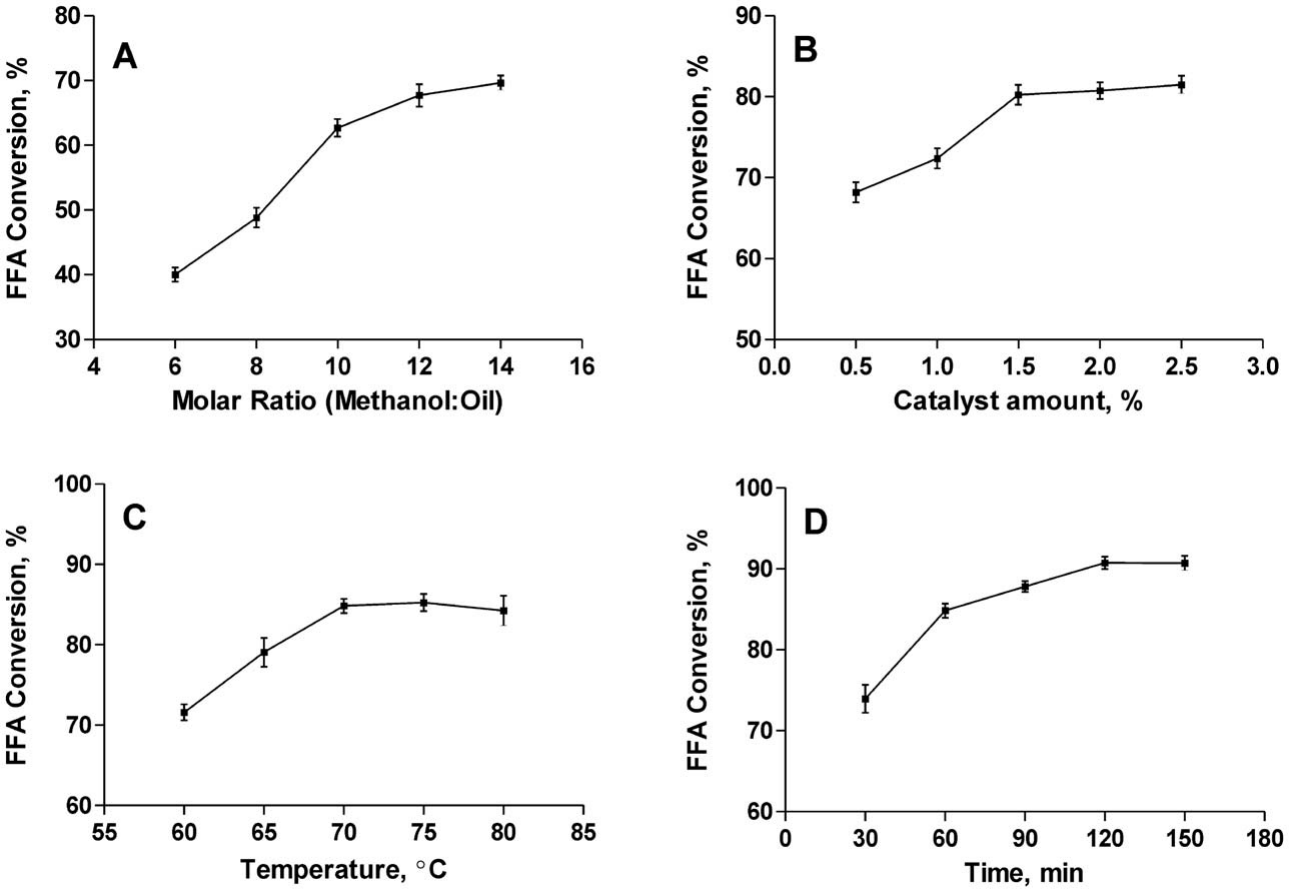
Optimization of four esterification parameters. A) methanol to oil molar ratio (1.0% catalyst, 65°C, 60 min, 200 rpm); B) catalyst amount (12∶1 methanol to oil molar ratio, 65°C, 60 min, 200 rpm); C) temperature (1.5% catalyst, 12∶1methanol to oil molar ratio, 60 min, 200 rpm); D) reaction time (1.5% catalyst, 12∶1methanol to oil molar ratio, 70°C, 200 rpm). Each experiment was repeated for three times.

### Optimization of Parameters Affecting Biodiesel Production

Optimized in this study for FFA acid-esterification are four parameters: methanol to oil molar ratio, catalyst amount, reaction temperature and reaction time.

Fig. 4A shows the effect of the methanol to oil molar ratio on FFA conversion. The results indicate that the higher the ratio, the higher the conversion rate. A molar ratio less than 12∶1 resulted in a lower conversion rate, while a ratio more than 12∶1 could not improve the conversion significantly. Hence, the methanol to oil molar ratio 12∶1 was selected for esterification. A higher methanol to fatty acid ratio could result in increased ester formation, but could also interfere with the separation of glycerin in later stages [9].

**Table 3:** Properties of *B. peregrine* larvae oil-based biodiesel and three known biodiesels.

Properties	Units	ASTM	*B. peregrine*	*H. illucens* a	*C. megacephala* b	Rapeseed c
Density 15°C	kg/m^3^	n/a	884.2	885	874.3	880
Viscosity at 40°C	mm^2^/s	1.9–6.0	5.6	5.8	4.0	6.35
Ester content	%	n/a	98.6	97.2	n/a	n/a
Water	%	0.05	<0.03	0.03	<0.03	0.03
Flash point	°C	130	146	123	170	n/a
Cetane number		47	52	53	54.8	45
Acid value	mg KOH/g	0.8	0.62	1.1	0.35	0.3
Distillation temperature	°C	360	323	360	337.0	352

n/a stands for not reported;

a data from literature [10];

b data from literature [11];

c data from literature [20].

Fig. 4B shows the effect of catalyst concentration on the FFA acid-esterification. The FFA conversion rate was very low (72.38%) at a catalyst concentration of 1.0%, but dramatically increased to 80.26% at 1.5%. At a concentration of more than 1.5%, however, the conversion rate did not increase significantly. Therefore, 1.5% was determined as the “optimal” catalyst concentration, because a lower amount of acid catalyst is not able to reduce the acid value of the reactants to the desired limit, whereas a higher amount will result in the darkening of the product [17].

Fig. 4C depicts the effect of temperature on FFA conversion. It was found that the temperature has a positive influence on FFA conversion, which could be attributed to the mass transfer efficiency, a high solubility of crude fat required for efficient mass transfer [24]. However, when the temperature was higher than 70°C, the conversion improvement was not significant. Therefore, 70°C was selected as the optimal esterification temperature.

The effect of reaction time on the FFA conversion rate is presented in Fig. 4D. Among the five sets of reaction time: 30 min, 60 min, 90 min, 120 min and 150 min, 120 min was selected for the optimal esterification time, because FFA conversion rate increased dramatically from 84.8% to 90.8% while the time was increased from 60 min to 120 min at a 30 min interval. However, a reaction time longer than 120 min did not increase the conversion significantly.

The above optimized parameters (the reaction temperature 70°C, the methanol to fat molar ratio 12∶1, the catalyst amount 1.5% and the reaction time 120 min) were applied for pretreatment of the crude oils extracted from the BPL grown on three types of solid organic wastes. After the pretreatment, the acid value of BPL oil was reduced to 1.75 mg KOH/g, and the usual acid value of feedstock for alkaline transesterification should be lower than 5 mg KOH/g [24]. The conversion rate of triglycerides (crude oil) to esters reached 92.3% by alkaline trans-esterification using 1% sodium hydroxide (NaOH) as catalyst [9].

### Fuel Properties

Table 3 shows the properties of BPL oil-based biodiesel in comparison to two other insect oil-based and one plant oil-based biodiesels. Most of the properties of the biodiesel from BPL fed on solid organic wastes have met the specifications of ASTM D6751, including density (884.2 kg/m^3^), viscosity (5.6 mm^2^/s), flash point (146°C), cetane number (52), ester content (98.6%) and acid value (0.62 mg KOH/g). Biodiesel properties are strongly influenced by the characteristics of individual fatty esters. The viscosity of BPL biodiesel is 5.6 mm^2^/s, higher than that of *C. megacephala* (4.0 mm^2^/s) biodiesel, but lower than that of *H. illucens* (5.8 mm^2^/s) and rapeseed (6.35 mm^2^/s) biodiesels. Viscosity affects the atomization of a fuel upon injection into the combustion chamber and ultimately the formation of engine deposits [25]. The flash point of BPL biodiesel (146°C) agrees well with the minimum specifications in ASTM D6751 (130°C), higher than that in *H. illucens* biodiesel (123°C). A higher flash point value means a higher handling and storing safety index. The cetane number is a dimensionless indicator related to the ignition quality of a fuel in a diesel engine. It is generally believed that the higher the cetane number, the better the ignition quality of a fuel, and vice versa [26]. The BPL biodiesel has a higher cetane number than ASTM D6751 (47) and rapeseed biodiesel (45), but lower than the other two insect oil-based biodiesels (53 and 54.8 in *H. illucens* and *C. megacephala,*), respectively. The cetane number increases with the increase of the chain length and saturation of a feedstock oil. It is worth noting that the BPL oil contains 44.5% oleic acid, nearly double that in *H. illucens* (23.6%) and *C. megacephala* (24.4%) oils. Knothe pointed that methyl oleate has been proposed as a suitable major component in biodiesel fuels for improving their properties [26].

### Conclusions

This study indicates that *Boettcherisca peregrine* holds a high promise for converting solid organic wastes into an alternative feedstock for biodiesel production due to its high oil content (19.5–31.1%) and short production cycle (4 days). The yields of BPL converted from swine manure, GFR, and SRF reach to 10.7%, 12.6% and 9.9% respectively. In addition, four parameters for acid-esterification of FFA in BPL oil were optimized: 12∶1 methanol to oil molar ratio, 1.5% catalyst at 70°C for 120 min, under which conditions, the conversion rate of FFA and triglycerides into biodiesel reached 90.8% and 92.3%, respectively. Compared with the other two insects (*H. illucens* and *C. Megacephala*) BPL oil contains more unsaturated fatty acid (75.1%) and Odd-carbon fatty acid (2.4%). The properties of the biodiesel from BPL oil met the standard ASTM D6751 including density (884.2 kg/m^3^), viscosity (5.6 mm^2^/s), flash point (146°C), cetane number (52) and ester contents (98.6%). The results of this study demonstrated that BPL can recycle different solid organic wastes into clean energy, and reduce environmental pollution of wastes.
